# Schizandrol A: a lignan from *Schisandra chinensis*

**DOI:** 10.1107/S1600536808021545

**Published:** 2008-07-16

**Authors:** Liang Zhao, Xiaoling Yu, Chungang Chen

**Affiliations:** a School of Food Science, Henan Institute of Science and Technology, Xinxiang 453003, People’s Republic of China

## Abstract

The title compound (systematic name: 1,2,3,10,11,12-hexa­meth­oxy-6,7-dimethyl-5,6,7,8-tetra­hydro­dibenzo[*a*,*c*]cyclo­oc­ten-6-ol), C_24_H_32_O_7_, has a dibenzocyclo­octa­diene skeleton. There are three mol­ecules in the asymmetric unit, which are related by a pseudo-translation in the direction of the *c* axis. Nevertheless, the three mol­ecules differ in the torsion angle of one of the meth­oxy groups. The dihedral angles between the two aromatic rings are 62.39 (10), 62.65 (10) and 61.84 (10)° for the three mol­ecules. The crystal packing is stabilized by a series of O—H⋯O and C—H⋯O hydrogen bonds, as well as C—H⋯π inter­actions.

## Related literature

For the pharmacology of schizandrol A, see Lee *et al.* (1999 ▶, 2003 ▶). For a similar structure, see: Wang *et al.* (2004 ▶).
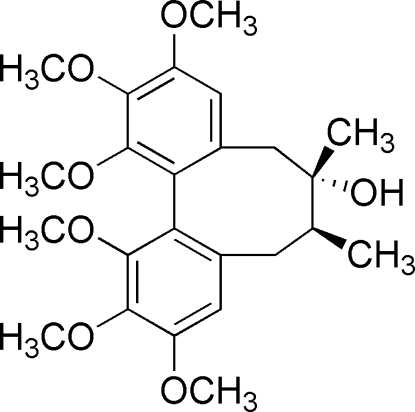


## Experimental

### 

#### Crystal data

C_24_H_32_O_7_
*M* *_r_* = 432.50
Orthorhombic, 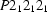

*a* = 16.084 (3) Å
*b* = 18.107 (4) Å
*c* = 23.229 (5) Å
*V* = 6765 (2) Å^3^
*Z* = 12
Mo *K*α radiation
μ = 0.09 mm^−1^
*T* = 113 (2) K
0.20 × 0.18 × 0.14 mm

#### Data collection

Rigaku Saturn CCD area-detector diffractometer
Absorption correction: multi-scan (*CrystalClear*; Rigaku/MSC, 2005 ▶) *T*_min_ = 0.982, *T*_max_ = 0.987
48456 measured reflections
8487 independent reflections
7852 reflections with *I* > 2σ(*I*)
*R*_int_ = 0.051

#### Refinement

*R*[*F* ^2^ > 2σ(*F* ^2^)] = 0.044
*wR*(*F* ^2^) = 0.110
*S* = 1.09
8487 reflections
865 parameters
H-atom parameters constrained
Δρ_max_ = 0.18 e Å^−3^
Δρ_min_ = −0.24 e Å^−3^

### 

Data collection: *CrystalClear* (Rigaku/MSC, 2005 ▶); cell refinement: *CrystalClear*; data reduction: *CrystalClear*; program(s) used to solve structure: *SHELXS97* (Sheldrick, 2008 ▶); program(s) used to refine structure: *SHELXL97* (Sheldrick, 2008 ▶); molecular graphics: *SHELXTL* (Sheldrick, 2008 ▶); software used to prepare material for publication: *CrystalStructure* (Rigaku/MSC, 2005 ▶).

## Supplementary Material

Crystal structure: contains datablocks global, I. DOI: 10.1107/S1600536808021545/bt2738sup1.cif

Structure factors: contains datablocks I. DOI: 10.1107/S1600536808021545/bt2738Isup2.hkl

Additional supplementary materials:  crystallographic information; 3D view; checkCIF report

## Figures and Tables

**Table 1:** Selected torsion angles (°)

C7—O3—C2—C3	98.2 (2)
C46—O11—C42—C43	46.7 (3)
C55—O17—C50—C51	93.0 (2)

**Table 2:** Hydrogen-bond geometry (Å, °) *Cg*1 is the centroid of the C49–C54 ring, *Cg*2 is the centroid of the C40–C45 ring and *Cg*3 is the centroid of the C1–C6 ring.

*D*—H⋯*A*	*D*—H	H⋯*A*	*D*⋯*A*	*D*—H⋯*A*
O7—H7⋯O2^i^	0.84	2.10	2.836 (2)	146
O14—H14⋯O16^ii^	0.84	2.14	2.889 (2)	149
O21—H21⋯O12^iii^	0.84	2.00	2.793 (2)	156
C11—H11⋯O3^i^	1.00	2.58	3.469 (3)	148
C37—H37*A*⋯O8^iv^	0.98	2.60	3.490 (3)	151
C37—H37*C*⋯O5^v^	0.98	2.58	3.498 (3)	157
C38—H38*B*⋯O18^ii^	0.98	2.51	3.389 (3)	150
C24—H24*B*⋯*Cg*1^ii^	0.98	2.80	3.710 (3)	156
C31—H31*B*⋯*Cg*2^vi^	0.98	2.71	3.653 (3)	181
C72—H72*B*⋯*Cg*3^ii^	0.98	2.94	3.894 (3)	164

Symmetry codes: (i) 
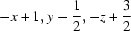
; (ii) 
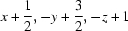
; (iii) 
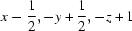
; (iv) 
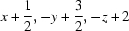
; (v) 
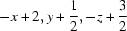
; (vi) 
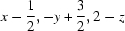
.

**Fig. 1.**
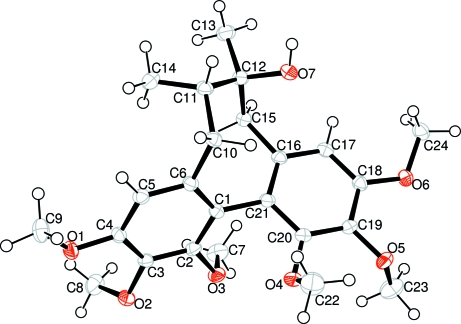
View of the first molecule in the asymmetric unit with the atom-numbering scheme and 50% probability displacement ellipsoids.

**Fig. 2.**
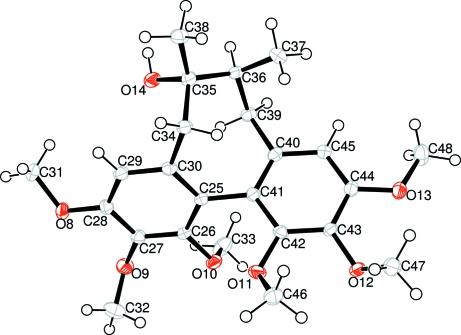
View of the second molecule in the asymmetric unit with the atom-numbering scheme and 50% probability displacement ellipsoids.

**Fig. 3.**
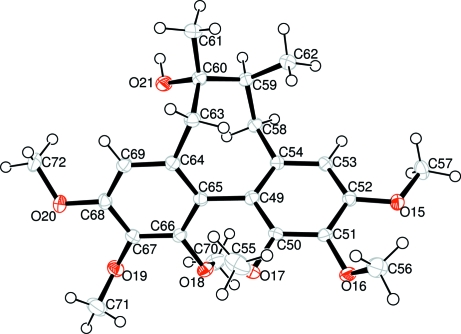
View of the third molecule in the asymmetric unit with the atom-numbering scheme and 50% probability displacement ellipsoids.

## supplementary crystallographic information

### Comment

Schizandrol A, with a special multi-pharmacological activity, has been found to have a relatively large potential in the application of cancer treatment (Lee *et al.*,  1999, 2003).

The title compound has the eight-membered ring in a twisted-boat-chair conformation. There are three molecules in the asymmetric unit, which are related by a pseudo-translation in direction of the c-axis. Nevertheless, the three molecules differ in the torsion angle of one of the methoxy groups (Table 1).

For the first molecule (Fig. 1), the two benzene rings form a dihedral angle of 62.39 (10)°, for the second (Fig. 2), the two benzene rings form an angle of 62.65 (10)°, and for the third (Fig. 3), the two benzene rings form an angle of 61.84 (10)°.

The crystal packing is stabilized by O—H···O and C—H···O hydrogen bonds (Table 1) as well as C—H···π interactions (H24B···*Cg*1^i^   2.80Å, C24—H24B···*Cg*1^i^   156°, H31B···*Cg*2^ii^  2.71Å, C31—H31B···*Cg*2^ii^  161° H72B···*Cg*3^i^ 2.94Å, C72—H72B···*Cg*3^i^ 164°; symmetry operators: (i)   *x* + 1/2, -*y* + 1/2, -*z* + 1, (ii)  *x* - 1/2, -*y* + 3/2, -*z* + 2; *Cg*1 is the centroid of the ring composed of C49—C54, *Cg*2 is the centroid of the ring composed of  C40—C45, *Cg*3 is the centroid of the ring composed of  C1—C6 ring).

### Experimental

Schizandrol A was isolated from  Schisandra chinensis. Crystals were obtained by   evaporation of a methanolic solution.

### Refinement

In the absence of anomalous scatterers Friedel pairs were merged and the absolute configuration was set according to the literature. All H atoms were positioned geometrically and refined as riding atoms, with U(H) = 1.2 U_eq_(CH and CH_2_) and with C—H ranging from 0.95 to 1.0 Å and U(H) = 1.5 U_eq_(CH_3_ and O) with C-H = 0.99Å and O-H = 0.84Å. The methyl and hydroxyl groups were allowed to rotate but not to tip.

### Crystal data

**Table:**

C_24_H_32_O_7_	*F*_000_ = 2784
*M**_r_* = 432.50	*D*_x_ = 1.274 Mg m^−^^3^
Orthorhombic, *P*2_1_2_1_2_1_	Mo *K*α radiation λ = 0.71073 Å
Hall symbol: P 2ac 2ab	Cell parameters from 18705 reflections
*a* = 16.084 (3) Å	θ = 1.5–27.6º
*b* = 18.107 (4) Å	µ = 0.09 mm^−^^1^
*c* = 23.229 (5) Å	*T* = 113 (2) K
*V* = 6765 (2) Å^3^	Block, colourless
*Z* = 12	0.20 × 0.18 × 0.14 mm

### Data collection

**Table:**

Rigaku Saturn CCD area-detector diffractometer	8487 independent reflections
Radiation source: rotating anode	7852 reflections with *I* > 2σ(*I*)
Monochromator: confocal	*R*_int_ = 0.051
Detector resolution: 7.31 pixels mm^-1^	θ_max_ = 27.5º
*T* = 113(2) K	θ_min_ = 1.5º
ω scans	*h* = −20→17
Absorption correction: multi-scan(CrystalClear; Rigaku/MSC, 2005)	*k* = −23→21
*T*_min_ = 0.982, *T*_max_ = 0.987	*l* = −30→27
48456 measured reflections	

### Refinement

**Table:**

Refinement on *F*^2^	Secondary atom site location: difference Fourier map
Least-squares matrix: full	Hydrogen site location: inferred from neighbouring sites
*R*[*F*^2^ > 2σ(*F*^2^)] = 0.044	H-atom parameters constrained
*wR*(*F*^2^) = 0.110	*w* = 1/[σ^2^(*F*_o_^2^) + (0.0626*P*)^2^ + 0.2401*P*] where *P* = (*F*_o_^2^ + 2*F*_c_^2^)/3
*S* = 1.09	(Δ/σ)_max_ = 0.001
8487 reflections	Δρ_max_ = 0.18 e Å^−^^3^
865 parameters	Δρ_min_ = −0.24 e Å^−^^3^
Primary atom site location: structure-invariant direct methods	Extinction correction: none

### Special details

**Table:**

Geometry. All e.s.d.'s (except the e.s.d. in the dihedral angle between two l.s. planes) are estimated using the full covariance matrix. The cell e.s.d.'s are taken into account individually in the estimation of e.s.d.'s in distances, angles and torsion angles; correlations between e.s.d.'s in cell parameters are only used when they are defined by crystal symmetry. An approximate (isotropic) treatment of cell e.s.d.'s is used for estimating e.s.d.'s involving l.s. planes.
Refinement. Refinement of *F*^2^ against ALL reflections. The weighted *R*-factor *wR* and goodness of fit *S* are based on *F*^2^, conventional *R*-factors *R* are based on *F*, with *F* set to zero for negative *F*^2^. The threshold expression of *F*^2^ > σ(*F*^2^) is used only for calculating *R*-factors(gt) *etc*. and is not relevant to the choice of reflections for refinement. *R*-factors based on *F*^2^ are statistically about twice as large as those based on *F*, and *R*- factors based on ALL data will be even larger.

### Fractional atomic coordinates and isotropic or equivalent isotropic displacement parameters (Å^2^)

**Table:**

	*x*	*y*	*z*	*U*_iso_*/*U*_eq_	
O1	0.22101 (9)	0.35381 (8)	0.73077 (7)	0.0232 (3)	
O2	0.32692 (9)	0.45994 (8)	0.76569 (7)	0.0239 (3)	
O3	0.49211 (9)	0.43468 (8)	0.77143 (6)	0.0230 (3)	
O4	0.58793 (9)	0.39176 (9)	0.66990 (7)	0.0291 (4)	
O5	0.75662 (9)	0.37642 (9)	0.67774 (7)	0.0292 (4)	
O6	0.82536 (9)	0.27751 (9)	0.74939 (7)	0.0237 (3)	
O7	0.60082 (9)	0.09979 (8)	0.75711 (7)	0.0249 (3)	
H7	0.6008	0.0541	0.7509	0.037*	
O8	0.67403 (9)	0.71666 (9)	0.91986 (7)	0.0227 (3)	
O9	0.74232 (9)	0.60393 (9)	0.86118 (7)	0.0255 (3)	
O10	0.90954 (9)	0.57743 (8)	0.86623 (6)	0.0215 (3)	
O11	1.00471 (9)	0.55230 (8)	0.96847 (7)	0.0248 (4)	
O12	1.17213 (9)	0.51920 (8)	0.94694 (7)	0.0221 (3)	
O13	1.27229 (9)	0.62086 (9)	0.90144 (7)	0.0244 (3)	
O14	0.89486 (9)	0.88450 (9)	0.92657 (7)	0.0234 (3)	
H14	0.8960	0.9291	0.9165	0.035*	
O15	0.22728 (9)	0.36321 (8)	0.07007 (7)	0.0221 (3)	
O16	0.33017 (9)	0.47290 (8)	0.10673 (6)	0.0215 (3)	
O17	0.49504 (9)	0.44981 (8)	0.11691 (6)	0.0205 (3)	
O18	0.59386 (9)	0.41675 (8)	0.01820 (6)	0.0217 (3)	
O19	0.76177 (9)	0.39393 (9)	0.01571 (7)	0.0259 (3)	
O20	0.83295 (9)	0.29020 (9)	0.08307 (7)	0.0243 (3)	
O21	0.61530 (9)	0.11886 (9)	0.08946 (7)	0.0261 (3)	
H21	0.6191	0.0734	0.0828	0.039*	
C1	0.47537 (13)	0.30913 (12)	0.73820 (9)	0.0200 (4)	
C2	0.44148 (13)	0.37730 (12)	0.75383 (9)	0.0197 (4)	
C3	0.35660 (13)	0.39076 (11)	0.75195 (9)	0.0192 (4)	
C4	0.30337 (12)	0.33497 (12)	0.73236 (9)	0.0189 (4)	
C5	0.33611 (13)	0.26724 (12)	0.71663 (9)	0.0201 (4)	
H5	0.2999	0.2296	0.7032	0.024*	
C6	0.42133 (13)	0.25307 (12)	0.72008 (9)	0.0189 (4)	
C7	0.51498 (16)	0.42987 (14)	0.83038 (10)	0.0317 (6)	
H7A	0.5510	0.3869	0.8361	0.048*	
H7B	0.4649	0.4246	0.8540	0.048*	
H7C	0.5448	0.4748	0.8417	0.048*	
C8	0.28384 (15)	0.46474 (14)	0.81940 (11)	0.0339 (6)	
H8A	0.3229	0.4558	0.8510	0.051*	
H8B	0.2395	0.4276	0.8205	0.051*	
H8C	0.2596	0.5141	0.8235	0.051*	
C9	0.16586 (13)	0.30434 (14)	0.70256 (10)	0.0279 (5)	
H9A	0.1660	0.2568	0.7227	0.042*	
H9B	0.1840	0.2972	0.6627	0.042*	
H9C	0.1095	0.3250	0.7029	0.042*	
C10	0.45252 (13)	0.17746 (11)	0.70287 (9)	0.0205 (4)	
H10A	0.5102	0.1827	0.6885	0.025*	
H10B	0.4181	0.1595	0.6704	0.025*	
C11	0.45200 (13)	0.11750 (12)	0.75076 (9)	0.0213 (4)	
H11	0.4556	0.0694	0.7298	0.026*	
C12	0.52938 (13)	0.11905 (12)	0.79049 (9)	0.0204 (4)	
C13	0.51924 (14)	0.06450 (13)	0.84006 (10)	0.0276 (5)	
H13A	0.5728	0.0579	0.8595	0.041*	
H13B	0.5001	0.0169	0.8250	0.041*	
H13C	0.4783	0.0837	0.8675	0.041*	
C14	0.36875 (13)	0.11516 (14)	0.78232 (11)	0.0285 (5)	
H14A	0.3634	0.1590	0.8068	0.043*	
H14B	0.3661	0.0706	0.8062	0.043*	
H14C	0.3234	0.1144	0.7542	0.043*	
C15	0.54875 (13)	0.19628 (12)	0.81623 (9)	0.0201 (4)	
H15A	0.4954	0.2217	0.8239	0.024*	
H15B	0.5770	0.1892	0.8537	0.024*	
C16	0.60180 (13)	0.24614 (11)	0.77951 (9)	0.0193 (4)	
C17	0.68839 (13)	0.23942 (12)	0.78333 (9)	0.0201 (4)	
H17	0.7117	0.2044	0.8091	0.024*	
C18	0.74031 (13)	0.28263 (12)	0.75035 (9)	0.0204 (4)	
C19	0.70630 (13)	0.33534 (12)	0.71306 (9)	0.0211 (4)	
C20	0.62032 (13)	0.34205 (12)	0.70857 (10)	0.0224 (4)	
C21	0.56694 (13)	0.29808 (12)	0.74200 (9)	0.0201 (4)	
C22	0.59403 (17)	0.36666 (18)	0.61177 (10)	0.0438 (7)	
H22A	0.5638	0.3200	0.6076	0.066*	
H22B	0.6526	0.3591	0.6018	0.066*	
H22C	0.5699	0.4037	0.5860	0.066*	
C23	0.78962 (18)	0.44071 (16)	0.70428 (15)	0.0513 (8)	
H23A	0.8286	0.4263	0.7347	0.077*	
H23B	0.7442	0.4697	0.7210	0.077*	
H23C	0.8188	0.4705	0.6754	0.077*	
C24	0.86011 (14)	0.21001 (13)	0.76977 (10)	0.0270 (5)	
H24A	0.8310	0.1683	0.7520	0.040*	
H24B	0.8540	0.2073	0.8117	0.040*	
H24C	0.9192	0.2080	0.7597	0.040*	
C25	0.93024 (13)	0.68169 (11)	0.92894 (9)	0.0180 (4)	
C26	0.87680 (13)	0.63516 (11)	0.89785 (9)	0.0180 (4)	
C27	0.79197 (13)	0.64683 (12)	0.89631 (9)	0.0198 (4)	
C28	0.75777 (13)	0.70615 (12)	0.92637 (9)	0.0191 (4)	
C29	0.80935 (13)	0.75110 (12)	0.95864 (9)	0.0189 (4)	
H29	0.7858	0.7901	0.9806	0.023*	
C30	0.89546 (13)	0.74027 (11)	0.95958 (9)	0.0183 (4)	
C31	0.64328 (14)	0.78833 (13)	0.93384 (10)	0.0262 (5)	
H31A	0.6783	0.8259	0.9156	0.039*	
H31B	0.6443	0.7951	0.9757	0.039*	
H31C	0.5861	0.7933	0.9198	0.039*	
C32	0.71267 (17)	0.53897 (15)	0.88860 (12)	0.0400 (6)	
H32A	0.6740	0.5525	0.9194	0.060*	
H32B	0.7597	0.5116	0.9049	0.060*	
H32C	0.6839	0.5079	0.8604	0.060*	
C33	0.91734 (17)	0.59558 (14)	0.80678 (10)	0.0336 (6)	
H33A	0.9543	0.6383	0.8025	0.050*	
H33B	0.8625	0.6076	0.7910	0.050*	
H33C	0.9406	0.5534	0.7859	0.050*	
C34	0.94773 (13)	0.79501 (12)	0.99228 (9)	0.0195 (4)	
H34A	1.0010	0.7710	1.0025	0.023*	
H34B	0.9188	0.8072	1.0287	0.023*	
C35	0.96723 (13)	0.86801 (12)	0.96016 (9)	0.0204 (4)	
C36	1.04299 (13)	0.86312 (12)	0.91930 (9)	0.0202 (4)	
H36	1.0393	0.9079	0.8943	0.024*	
C37	1.12733 (13)	0.86895 (13)	0.94948 (10)	0.0257 (5)	
H37A	1.1323	0.8295	0.9782	0.039*	
H37B	1.1318	0.9170	0.9686	0.039*	
H37C	1.1719	0.8640	0.9210	0.039*	
C38	0.97951 (14)	0.92885 (13)	1.00476 (10)	0.0259 (5)	
H38A	0.9268	0.9384	1.0245	0.039*	
H38B	0.9983	0.9740	0.9855	0.039*	
H38C	1.0214	0.9132	1.0329	0.039*	
C39	1.03961 (13)	0.79643 (12)	0.87733 (9)	0.0201 (4)	
H39A	0.9810	0.7890	0.8658	0.024*	
H39B	1.0711	0.8098	0.8422	0.024*	
C40	1.07303 (13)	0.72303 (12)	0.89929 (9)	0.0190 (4)	
C41	1.02178 (12)	0.66995 (12)	0.92481 (9)	0.0175 (4)	
C42	1.05705 (12)	0.60297 (12)	0.94374 (9)	0.0193 (4)	
C43	1.14118 (12)	0.58866 (11)	0.93495 (9)	0.0187 (4)	
C44	1.19131 (12)	0.64162 (12)	0.90858 (9)	0.0198 (4)	
C45	1.15784 (13)	0.70844 (12)	0.89145 (9)	0.0195 (4)	
H45	1.1926	0.7447	0.8743	0.023*	
C46	1.03229 (14)	0.51599 (13)	1.01966 (10)	0.0270 (5)	
H46A	1.0638	0.5508	1.0434	0.041*	
H46B	1.0679	0.4742	1.0093	0.041*	
H46C	0.9840	0.4981	1.0413	0.041*	
C47	1.23501 (14)	0.51537 (14)	0.99111 (11)	0.0316 (6)	
H47A	1.2090	0.5213	1.0290	0.047*	
H47B	1.2758	0.5548	0.9851	0.047*	
H47C	1.2630	0.4674	0.9892	0.047*	
C48	1.32394 (14)	0.66689 (15)	0.86767 (10)	0.0311 (5)	
H48A	1.3263	0.7162	0.8849	0.047*	
H48B	1.3012	0.6705	0.8286	0.047*	
H48C	1.3800	0.6459	0.8660	0.047*	
C49	0.48299 (13)	0.32529 (12)	0.08063 (9)	0.0184 (4)	
C50	0.44632 (12)	0.39210 (12)	0.09731 (9)	0.0175 (4)	
C51	0.36115 (13)	0.40387 (11)	0.09355 (9)	0.0183 (4)	
C52	0.31019 (12)	0.34727 (12)	0.07249 (9)	0.0186 (4)	
C53	0.34539 (13)	0.28111 (12)	0.05587 (9)	0.0193 (4)	
H53	0.3107	0.2431	0.0412	0.023*	
C54	0.43121 (13)	0.26873 (12)	0.06014 (9)	0.0183 (4)	
C55	0.50873 (17)	0.44691 (16)	0.17689 (10)	0.0373 (6)	
H55A	0.5417	0.4030	0.1862	0.056*	
H55B	0.4552	0.4444	0.1969	0.056*	
H55C	0.5388	0.4913	0.1891	0.056*	
C56	0.27811 (15)	0.47761 (14)	0.15661 (11)	0.0321 (6)	
H56A	0.3097	0.4624	0.1907	0.048*	
H56B	0.2299	0.4451	0.1518	0.048*	
H56C	0.2591	0.5286	0.1615	0.048*	
C57	0.17460 (13)	0.31027 (14)	0.04303 (10)	0.0264 (5)	
H57A	0.1774	0.2635	0.0641	0.040*	
H57B	0.1929	0.3025	0.0033	0.040*	
H57C	0.1172	0.3284	0.0431	0.040*	
C58	0.46477 (13)	0.19487 (12)	0.04038 (9)	0.0211 (4)	
H58A	0.5224	0.2024	0.0266	0.025*	
H58B	0.4313	0.1784	0.0070	0.025*	
C59	0.46560 (13)	0.13179 (12)	0.08538 (10)	0.0221 (4)	
H59	0.4698	0.0852	0.0625	0.026*	
C60	0.54366 (13)	0.13266 (12)	0.12440 (9)	0.0222 (4)	
C61	0.53711 (15)	0.07272 (13)	0.17034 (11)	0.0300 (5)	
H61A	0.5906	0.0677	0.1901	0.045*	
H61B	0.5224	0.0257	0.1521	0.045*	
H61C	0.4941	0.0863	0.1983	0.045*	
C62	0.38356 (13)	0.12603 (13)	0.11798 (10)	0.0268 (5)	
H62A	0.3372	0.1288	0.0907	0.040*	
H62B	0.3794	0.1668	0.1456	0.040*	
H62C	0.3814	0.0789	0.1386	0.040*	
C63	0.55992 (13)	0.20784 (12)	0.15394 (9)	0.0215 (4)	
H63A	0.5057	0.2311	0.1629	0.026*	
H63B	0.5888	0.1988	0.1909	0.026*	
C64	0.61102 (13)	0.26150 (12)	0.11891 (9)	0.0190 (4)	
C65	0.57462 (13)	0.31591 (12)	0.08451 (9)	0.0183 (4)	
C66	0.62727 (13)	0.36206 (12)	0.05202 (9)	0.0186 (4)	
C67	0.71315 (13)	0.35299 (12)	0.05239 (9)	0.0197 (4)	
C68	0.74847 (13)	0.29777 (12)	0.08662 (9)	0.0207 (4)	
C69	0.69748 (13)	0.25376 (12)	0.12022 (9)	0.0198 (4)	
H69	0.7217	0.2176	0.1446	0.024*	
C70	0.58397 (17)	0.39464 (14)	−0.04001 (10)	0.0320 (6)	
H70A	0.5502	0.3496	−0.0416	0.048*	
H70B	0.6387	0.3850	−0.0570	0.048*	
H70C	0.5562	0.4340	−0.0616	0.048*	
C71	0.79704 (17)	0.45794 (15)	0.04198 (12)	0.0389 (6)	
H71A	0.8335	0.4429	0.0737	0.058*	
H71B	0.7524	0.4894	0.0569	0.058*	
H71C	0.8294	0.4854	0.0134	0.058*	
C72	0.86633 (14)	0.22107 (13)	0.10102 (11)	0.0286 (5)	
H72A	0.8353	0.1809	0.0827	0.043*	
H72B	0.8618	0.2167	0.1430	0.043*	
H72C	0.9249	0.2180	0.0897	0.043*	

### Atomic displacement parameters (Å^2^)

**Table:**

	*U*^11^	*U*^22^	*U*^33^	*U*^12^	*U*^13^	*U*^23^
O1	0.0161 (7)	0.0227 (8)	0.0309 (8)	0.0009 (6)	−0.0032 (6)	−0.0025 (7)
O2	0.0243 (8)	0.0170 (8)	0.0305 (8)	0.0024 (6)	0.0019 (7)	−0.0013 (7)
O3	0.0228 (7)	0.0172 (8)	0.0291 (8)	−0.0053 (6)	−0.0044 (7)	0.0007 (6)
O4	0.0250 (8)	0.0254 (9)	0.0369 (9)	0.0034 (7)	−0.0004 (7)	0.0144 (7)
O5	0.0214 (8)	0.0282 (9)	0.0379 (9)	−0.0047 (7)	0.0035 (7)	0.0105 (8)
O6	0.0158 (7)	0.0260 (8)	0.0293 (8)	0.0013 (6)	−0.0026 (6)	0.0044 (7)
O7	0.0253 (8)	0.0178 (8)	0.0315 (8)	0.0044 (6)	0.0042 (7)	−0.0003 (7)
O8	0.0158 (7)	0.0242 (8)	0.0283 (8)	0.0024 (6)	0.0002 (6)	−0.0022 (7)
O9	0.0238 (8)	0.0236 (8)	0.0292 (8)	−0.0030 (7)	−0.0066 (7)	−0.0053 (7)
O10	0.0233 (7)	0.0171 (8)	0.0240 (7)	0.0023 (6)	−0.0018 (6)	−0.0037 (6)
O11	0.0221 (8)	0.0174 (8)	0.0351 (9)	−0.0041 (6)	−0.0058 (7)	0.0081 (7)
O12	0.0209 (7)	0.0137 (7)	0.0317 (8)	0.0016 (6)	−0.0055 (7)	0.0003 (6)
O13	0.0188 (7)	0.0236 (8)	0.0307 (8)	0.0036 (6)	0.0025 (6)	0.0043 (7)
O14	0.0230 (8)	0.0174 (8)	0.0299 (8)	0.0026 (6)	−0.0043 (7)	0.0016 (7)
O15	0.0166 (7)	0.0203 (8)	0.0295 (8)	0.0003 (6)	−0.0016 (6)	−0.0024 (7)
O16	0.0233 (7)	0.0151 (7)	0.0262 (8)	0.0024 (6)	0.0040 (6)	−0.0007 (6)
O17	0.0225 (7)	0.0164 (7)	0.0225 (7)	−0.0044 (6)	−0.0003 (6)	−0.0017 (6)
O18	0.0243 (7)	0.0202 (8)	0.0205 (7)	0.0029 (6)	−0.0005 (6)	0.0034 (6)
O19	0.0233 (8)	0.0271 (9)	0.0273 (8)	−0.0037 (7)	0.0027 (7)	0.0038 (7)
O20	0.0160 (7)	0.0262 (8)	0.0306 (8)	0.0032 (6)	−0.0009 (6)	0.0009 (7)
O21	0.0235 (8)	0.0176 (8)	0.0373 (9)	0.0032 (6)	0.0048 (7)	−0.0010 (7)
C1	0.0186 (10)	0.0193 (11)	0.0221 (10)	0.0009 (8)	−0.0016 (8)	0.0034 (9)
C2	0.0212 (10)	0.0168 (10)	0.0212 (10)	−0.0039 (8)	−0.0017 (8)	0.0017 (9)
C3	0.0215 (10)	0.0143 (10)	0.0217 (10)	−0.0006 (8)	0.0006 (8)	0.0007 (8)
C4	0.0167 (9)	0.0208 (11)	0.0191 (9)	0.0011 (8)	−0.0018 (8)	0.0013 (9)
C5	0.0206 (10)	0.0178 (10)	0.0218 (10)	−0.0023 (8)	−0.0026 (8)	0.0000 (8)
C6	0.0204 (10)	0.0174 (10)	0.0190 (10)	0.0015 (8)	0.0008 (8)	0.0003 (8)
C7	0.0370 (13)	0.0292 (13)	0.0288 (12)	−0.0094 (11)	−0.0119 (11)	0.0005 (10)
C8	0.0348 (13)	0.0309 (13)	0.0359 (13)	−0.0009 (11)	0.0128 (11)	−0.0080 (11)
C9	0.0197 (10)	0.0358 (13)	0.0281 (11)	−0.0012 (9)	−0.0023 (9)	−0.0113 (10)
C10	0.0218 (10)	0.0192 (11)	0.0206 (10)	0.0023 (8)	0.0009 (9)	−0.0013 (8)
C11	0.0236 (10)	0.0144 (10)	0.0259 (10)	0.0000 (8)	−0.0017 (9)	−0.0011 (9)
C12	0.0220 (10)	0.0179 (10)	0.0212 (10)	0.0010 (8)	0.0009 (9)	0.0018 (9)
C13	0.0290 (12)	0.0225 (12)	0.0313 (12)	−0.0027 (9)	−0.0024 (10)	0.0065 (10)
C14	0.0250 (11)	0.0261 (12)	0.0345 (12)	−0.0048 (9)	0.0008 (10)	0.0059 (10)
C15	0.0206 (10)	0.0188 (10)	0.0210 (10)	0.0006 (8)	0.0004 (8)	0.0002 (9)
C16	0.0220 (10)	0.0138 (10)	0.0222 (10)	0.0002 (8)	−0.0006 (8)	−0.0018 (8)
C17	0.0222 (10)	0.0186 (10)	0.0195 (10)	0.0025 (8)	−0.0029 (8)	0.0007 (8)
C18	0.0181 (10)	0.0189 (10)	0.0243 (10)	0.0007 (8)	−0.0027 (9)	−0.0016 (9)
C19	0.0207 (10)	0.0178 (10)	0.0248 (10)	−0.0020 (8)	0.0009 (9)	0.0025 (9)
C20	0.0228 (10)	0.0165 (10)	0.0279 (11)	0.0010 (8)	0.0004 (9)	0.0042 (9)
C21	0.0191 (10)	0.0155 (10)	0.0258 (11)	0.0000 (8)	−0.0024 (8)	0.0018 (9)
C22	0.0406 (15)	0.0576 (19)	0.0330 (13)	0.0066 (14)	−0.0050 (12)	0.0194 (13)
C23	0.0438 (16)	0.0314 (15)	0.079 (2)	−0.0165 (13)	0.0047 (16)	0.0067 (16)
C24	0.0217 (11)	0.0276 (12)	0.0316 (12)	0.0047 (9)	−0.0041 (9)	0.0022 (10)
C25	0.0179 (10)	0.0135 (10)	0.0227 (10)	0.0006 (8)	−0.0001 (8)	0.0024 (8)
C26	0.0207 (10)	0.0129 (10)	0.0204 (10)	0.0011 (8)	−0.0001 (8)	0.0002 (8)
C27	0.0203 (10)	0.0183 (10)	0.0210 (10)	−0.0007 (8)	−0.0012 (8)	−0.0014 (8)
C28	0.0177 (10)	0.0209 (11)	0.0187 (10)	0.0001 (8)	0.0004 (8)	0.0034 (8)
C29	0.0213 (10)	0.0151 (10)	0.0203 (10)	0.0005 (8)	0.0012 (8)	−0.0010 (8)
C30	0.0212 (10)	0.0155 (10)	0.0180 (9)	−0.0004 (8)	−0.0012 (8)	0.0017 (8)
C31	0.0193 (10)	0.0247 (12)	0.0346 (12)	0.0061 (9)	0.0017 (9)	−0.0022 (10)
C32	0.0373 (14)	0.0304 (14)	0.0524 (17)	−0.0146 (11)	−0.0016 (13)	−0.0042 (13)
C33	0.0456 (15)	0.0306 (13)	0.0247 (11)	0.0133 (11)	0.0032 (11)	−0.0012 (10)
C34	0.0206 (10)	0.0165 (10)	0.0215 (10)	0.0000 (8)	−0.0010 (8)	−0.0002 (8)
C35	0.0210 (10)	0.0175 (10)	0.0228 (10)	0.0005 (8)	−0.0005 (8)	−0.0016 (9)
C36	0.0228 (10)	0.0134 (10)	0.0246 (10)	0.0005 (8)	0.0015 (9)	0.0014 (8)
C37	0.0221 (11)	0.0234 (12)	0.0316 (12)	−0.0053 (9)	0.0000 (9)	−0.0004 (10)
C38	0.0276 (11)	0.0198 (11)	0.0302 (12)	−0.0039 (9)	0.0012 (9)	−0.0061 (10)
C39	0.0230 (10)	0.0169 (10)	0.0203 (10)	0.0014 (9)	−0.0019 (8)	0.0015 (8)
C40	0.0220 (10)	0.0166 (10)	0.0185 (9)	0.0022 (8)	−0.0016 (8)	−0.0019 (8)
C41	0.0169 (9)	0.0165 (10)	0.0190 (9)	0.0001 (8)	−0.0023 (8)	−0.0008 (8)
C42	0.0177 (10)	0.0161 (10)	0.0242 (10)	−0.0033 (8)	−0.0040 (8)	0.0005 (9)
C43	0.0187 (10)	0.0133 (10)	0.0243 (10)	0.0022 (8)	−0.0035 (8)	−0.0002 (8)
C44	0.0179 (10)	0.0205 (11)	0.0209 (10)	0.0019 (8)	−0.0010 (8)	−0.0017 (9)
C45	0.0192 (10)	0.0174 (10)	0.0219 (10)	−0.0006 (8)	0.0008 (8)	0.0008 (9)
C46	0.0284 (12)	0.0242 (12)	0.0284 (11)	0.0023 (9)	−0.0003 (10)	0.0056 (10)
C47	0.0243 (11)	0.0264 (13)	0.0440 (14)	−0.0003 (9)	−0.0113 (11)	0.0089 (11)
C48	0.0225 (11)	0.0400 (15)	0.0308 (12)	0.0050 (10)	0.0047 (10)	0.0112 (11)
C49	0.0203 (10)	0.0184 (10)	0.0164 (9)	0.0007 (8)	−0.0010 (8)	0.0010 (8)
C50	0.0194 (10)	0.0147 (10)	0.0183 (9)	−0.0029 (8)	−0.0007 (8)	−0.0001 (8)
C51	0.0209 (10)	0.0151 (10)	0.0189 (10)	0.0017 (8)	0.0013 (8)	0.0022 (8)
C52	0.0175 (10)	0.0201 (11)	0.0181 (10)	0.0002 (8)	0.0004 (8)	0.0007 (8)
C53	0.0183 (10)	0.0188 (11)	0.0208 (10)	−0.0025 (8)	−0.0019 (8)	−0.0014 (8)
C54	0.0186 (10)	0.0174 (10)	0.0189 (9)	−0.0003 (8)	−0.0002 (8)	0.0006 (8)
C55	0.0488 (16)	0.0411 (15)	0.0221 (11)	−0.0188 (13)	−0.0064 (11)	−0.0029 (11)
C56	0.0343 (13)	0.0287 (13)	0.0333 (12)	0.0009 (10)	0.0114 (11)	−0.0056 (11)
C57	0.0178 (10)	0.0321 (13)	0.0294 (12)	−0.0030 (9)	−0.0025 (9)	−0.0066 (10)
C58	0.0215 (10)	0.0184 (11)	0.0233 (10)	0.0012 (8)	0.0015 (9)	−0.0025 (9)
C59	0.0210 (10)	0.0152 (10)	0.0300 (11)	0.0012 (8)	0.0002 (9)	−0.0018 (9)
C60	0.0212 (10)	0.0178 (11)	0.0275 (11)	0.0014 (8)	0.0018 (9)	0.0003 (9)
C61	0.0285 (12)	0.0228 (12)	0.0388 (13)	−0.0041 (10)	−0.0039 (10)	0.0099 (11)
C62	0.0238 (11)	0.0207 (11)	0.0360 (13)	−0.0022 (9)	0.0007 (10)	0.0006 (10)
C63	0.0222 (10)	0.0202 (11)	0.0220 (10)	0.0003 (8)	0.0017 (9)	0.0021 (9)
C64	0.0208 (10)	0.0179 (10)	0.0182 (10)	−0.0002 (8)	−0.0014 (8)	−0.0031 (8)
C65	0.0183 (10)	0.0171 (10)	0.0194 (10)	−0.0004 (8)	−0.0005 (8)	−0.0023 (8)
C66	0.0201 (10)	0.0161 (10)	0.0196 (10)	0.0018 (8)	−0.0010 (8)	−0.0006 (8)
C67	0.0198 (10)	0.0192 (11)	0.0202 (10)	−0.0020 (8)	0.0013 (8)	0.0001 (9)
C68	0.0171 (10)	0.0214 (11)	0.0237 (10)	0.0013 (8)	−0.0004 (8)	−0.0047 (9)
C69	0.0219 (10)	0.0178 (10)	0.0197 (10)	0.0013 (8)	−0.0028 (8)	−0.0020 (8)
C70	0.0443 (15)	0.0289 (13)	0.0227 (11)	0.0053 (11)	−0.0063 (10)	0.0015 (10)
C71	0.0367 (14)	0.0343 (15)	0.0456 (15)	−0.0152 (12)	−0.0042 (12)	0.0064 (12)
C72	0.0200 (11)	0.0292 (13)	0.0365 (12)	0.0074 (9)	−0.0045 (10)	0.0008 (11)

### Geometric parameters (Å, °)

**Table:**

O1—C4	1.368 (2)	C28—C29	1.383 (3)
O1—C9	1.421 (3)	C29—C30	1.399 (3)
O2—C3	1.378 (2)	C29—H29	0.9500
O2—C8	1.430 (3)	C30—C34	1.505 (3)
O3—C2	1.382 (2)	C31—H31A	0.9800
O3—C7	1.421 (3)	C31—H31B	0.9800
O4—C20	1.374 (3)	C31—H31C	0.9800
O4—C22	1.428 (3)	C32—H32A	0.9800
O5—C19	1.372 (3)	C32—H32B	0.9800
O5—C23	1.420 (3)	C32—H32C	0.9800
O6—C18	1.371 (2)	C33—H33A	0.9800
O6—C24	1.425 (3)	C33—H33B	0.9800
O7—C12	1.429 (2)	C33—H33C	0.9800
O7—H7	0.8400	C34—C35	1.550 (3)
O8—C28	1.369 (2)	C34—H34A	0.9900
O8—C31	1.426 (3)	C34—H34B	0.9900
O9—C27	1.381 (2)	C35—C38	1.525 (3)
O9—C32	1.420 (3)	C35—C36	1.547 (3)
O10—C26	1.382 (2)	C36—C37	1.530 (3)
O10—C33	1.425 (3)	C36—C39	1.553 (3)
O11—C42	1.371 (3)	C36—H36	1.0000
O11—C46	1.429 (3)	C37—H37A	0.9800
O12—C43	1.381 (2)	C37—H37B	0.9800
O12—C47	1.442 (3)	C37—H37C	0.9800
O13—C44	1.366 (2)	C38—H38A	0.9800
O13—C48	1.414 (3)	C38—H38B	0.9800
O14—C35	1.433 (2)	C38—H38C	0.9800
O14—H14	0.8400	C39—C40	1.522 (3)
O15—C52	1.366 (2)	C39—H39A	0.9900
O15—C57	1.425 (3)	C39—H39B	0.9900
O16—C51	1.380 (2)	C40—C41	1.398 (3)
O16—C56	1.432 (3)	C40—C45	1.401 (3)
O17—C50	1.383 (2)	C41—C42	1.409 (3)
O17—C55	1.411 (3)	C42—C43	1.393 (3)
O18—C66	1.373 (2)	C43—C44	1.395 (3)
O18—C70	1.419 (3)	C44—C45	1.383 (3)
O19—C67	1.374 (2)	C45—H45	0.9500
O19—C71	1.427 (3)	C46—H46A	0.9800
O20—C68	1.368 (2)	C46—H46B	0.9800
O20—C72	1.425 (3)	C46—H46C	0.9800
O21—C60	1.431 (3)	C47—H47A	0.9800
O21—H21	0.8400	C47—H47B	0.9800
C1—C2	1.397 (3)	C47—H47C	0.9800
C1—C6	1.401 (3)	C48—H48A	0.9800
C1—C21	1.489 (3)	C48—H48B	0.9800
C2—C3	1.388 (3)	C48—H48C	0.9800
C3—C4	1.400 (3)	C49—C50	1.400 (3)
C4—C5	1.384 (3)	C49—C54	1.403 (3)
C5—C6	1.397 (3)	C49—C65	1.486 (3)
C5—H5	0.9500	C50—C51	1.389 (3)
C6—C10	1.512 (3)	C51—C52	1.401 (3)
C7—H7A	0.9800	C52—C53	1.380 (3)
C7—H7B	0.9800	C53—C54	1.402 (3)
C7—H7C	0.9800	C53—H53	0.9500
C8—H8A	0.9800	C54—C58	1.514 (3)
C8—H8B	0.9800	C55—H55A	0.9800
C8—H8C	0.9800	C55—H55B	0.9800
C9—H9A	0.9800	C55—H55C	0.9800
C9—H9B	0.9800	C56—H56A	0.9800
C9—H9C	0.9800	C56—H56B	0.9800
C10—C11	1.554 (3)	C56—H56C	0.9800
C10—H10A	0.9900	C57—H57A	0.9800
C10—H10B	0.9900	C57—H57B	0.9800
C11—C14	1.527 (3)	C57—H57C	0.9800
C11—C12	1.550 (3)	C58—C59	1.549 (3)
C11—H11	1.0000	C58—H58A	0.9900
C12—C13	1.526 (3)	C58—H58B	0.9900
C12—C15	1.552 (3)	C59—C62	1.525 (3)
C13—H13A	0.9800	C59—C60	1.549 (3)
C13—H13B	0.9800	C59—H59	1.0000
C13—H13C	0.9800	C60—C61	1.526 (3)
C14—H14A	0.9800	C60—C63	1.547 (3)
C14—H14B	0.9800	C61—H61A	0.9800
C14—H14C	0.9800	C61—H61B	0.9800
C15—C16	1.507 (3)	C61—H61C	0.9800
C15—H15A	0.9900	C62—H62A	0.9800
C15—H15B	0.9900	C62—H62B	0.9800
C16—C21	1.399 (3)	C62—H62C	0.9800
C16—C17	1.401 (3)	C63—C64	1.510 (3)
C17—C18	1.377 (3)	C63—H63A	0.9900
C17—H17	0.9500	C63—H63B	0.9900
C18—C19	1.400 (3)	C64—C65	1.397 (3)
C19—C20	1.392 (3)	C64—C69	1.398 (3)
C20—C21	1.405 (3)	C65—C66	1.409 (3)
C22—H22A	0.9800	C66—C67	1.391 (3)
C22—H22B	0.9800	C67—C68	1.398 (3)
C22—H22C	0.9800	C68—C69	1.384 (3)
C23—H23A	0.9800	C69—H69	0.9500
C23—H23B	0.9800	C70—H70A	0.9800
C23—H23C	0.9800	C70—H70B	0.9800
C24—H24A	0.9800	C70—H70C	0.9800
C24—H24B	0.9800	C71—H71A	0.9800
C24—H24C	0.9800	C71—H71B	0.9800
C25—C30	1.394 (3)	C71—H71C	0.9800
C25—C26	1.404 (3)	C72—H72A	0.9800
C25—C41	1.491 (3)	C72—H72B	0.9800
C26—C27	1.381 (3)	C72—H72C	0.9800
C27—C28	1.394 (3)		
			
C4—O1—C9	117.36 (17)	C38—C35—C36	110.86 (18)
C3—O2—C8	115.17 (17)	O14—C35—C34	105.99 (17)
C2—O3—C7	113.05 (17)	C38—C35—C34	108.38 (17)
C20—O4—C22	112.56 (19)	C36—C35—C34	113.95 (17)
C19—O5—C23	113.9 (2)	C37—C36—C35	114.40 (18)
C18—O6—C24	116.35 (17)	C37—C36—C39	111.85 (18)
C12—O7—H7	109.5	C35—C36—C39	113.71 (17)
C28—O8—C31	116.27 (17)	C37—C36—H36	105.3
C27—O9—C32	113.27 (18)	C35—C36—H36	105.3
C26—O10—C33	111.97 (16)	C39—C36—H36	105.3
C42—O11—C46	117.76 (16)	C36—C37—H37A	109.5
C43—O12—C47	116.11 (16)	C36—C37—H37B	109.5
C44—O13—C48	117.73 (17)	H37A—C37—H37B	109.5
C35—O14—H14	109.5	C36—C37—H37C	109.5
C52—O15—C57	117.17 (17)	H37A—C37—H37C	109.5
C51—O16—C56	116.40 (17)	H37B—C37—H37C	109.5
C50—O17—C55	112.66 (17)	C35—C38—H38A	109.5
C66—O18—C70	112.67 (17)	C35—C38—H38B	109.5
C67—O19—C71	113.53 (18)	H38A—C38—H38B	109.5
C68—O20—C72	116.40 (17)	C35—C38—H38C	109.5
C60—O21—H21	109.5	H38A—C38—H38C	109.5
C2—C1—C6	118.42 (19)	H38B—C38—H38C	109.5
C2—C1—C21	119.29 (19)	C40—C39—C36	117.14 (17)
C6—C1—C21	122.29 (19)	C40—C39—H39A	108.0
O3—C2—C3	117.20 (19)	C36—C39—H39A	108.0
O3—C2—C1	120.73 (18)	C40—C39—H39B	108.0
C3—C2—C1	122.06 (19)	C36—C39—H39B	108.0
O2—C3—C2	119.56 (18)	H39A—C39—H39B	107.3
O2—C3—C4	121.28 (18)	C41—C40—C45	119.96 (19)
C2—C3—C4	119.02 (19)	C41—C40—C39	122.30 (18)
O1—C4—C5	125.60 (19)	C45—C40—C39	117.71 (19)
O1—C4—C3	114.89 (19)	C40—C41—C42	119.11 (18)
C5—C4—C3	119.51 (19)	C40—C41—C25	120.76 (19)
C4—C5—C6	121.4 (2)	C42—C41—C25	120.02 (19)
C4—C5—H5	119.3	O11—C42—C43	122.23 (19)
C6—C5—H5	119.3	O11—C42—C41	117.34 (18)
C5—C6—C1	119.54 (19)	C43—C42—C41	120.4 (2)
C5—C6—C10	118.47 (19)	O12—C43—C42	119.34 (19)
C1—C6—C10	121.98 (18)	O12—C43—C44	120.43 (18)
O3—C7—H7A	109.5	C42—C43—C44	119.88 (19)
O3—C7—H7B	109.5	O13—C44—C45	125.3 (2)
H7A—C7—H7B	109.5	O13—C44—C43	114.55 (19)
O3—C7—H7C	109.5	C45—C44—C43	120.20 (19)
H7A—C7—H7C	109.5	C44—C45—C40	120.4 (2)
H7B—C7—H7C	109.5	C44—C45—H45	119.8
O2—C8—H8A	109.5	C40—C45—H45	119.8
O2—C8—H8B	109.5	O11—C46—H46A	109.5
H8A—C8—H8B	109.5	O11—C46—H46B	109.5
O2—C8—H8C	109.5	H46A—C46—H46B	109.5
H8A—C8—H8C	109.5	O11—C46—H46C	109.5
H8B—C8—H8C	109.5	H46A—C46—H46C	109.5
O1—C9—H9A	109.5	H46B—C46—H46C	109.5
O1—C9—H9B	109.5	O12—C47—H47A	109.5
H9A—C9—H9B	109.5	O12—C47—H47B	109.5
O1—C9—H9C	109.5	H47A—C47—H47B	109.5
H9A—C9—H9C	109.5	O12—C47—H47C	109.5
H9B—C9—H9C	109.5	H47A—C47—H47C	109.5
C6—C10—C11	116.20 (17)	H47B—C47—H47C	109.5
C6—C10—H10A	108.2	O13—C48—H48A	109.5
C11—C10—H10A	108.2	O13—C48—H48B	109.5
C6—C10—H10B	108.2	H48A—C48—H48B	109.5
C11—C10—H10B	108.2	O13—C48—H48C	109.5
H10A—C10—H10B	107.4	H48A—C48—H48C	109.5
C14—C11—C12	114.76 (18)	H48B—C48—H48C	109.5
C14—C11—C10	111.57 (18)	C50—C49—C54	118.31 (19)
C12—C11—C10	114.15 (17)	C50—C49—C65	119.97 (19)
C14—C11—H11	105.1	C54—C49—C65	121.72 (19)
C12—C11—H11	105.1	O17—C50—C51	117.61 (19)
C10—C11—H11	105.1	O17—C50—C49	120.33 (18)
O7—C12—C13	109.72 (17)	C51—C50—C49	122.04 (19)
O7—C12—C11	108.55 (17)	O16—C51—C50	118.76 (18)
C13—C12—C11	110.60 (18)	O16—C51—C52	121.94 (18)
O7—C12—C15	105.51 (16)	C50—C51—C52	119.13 (19)
C13—C12—C15	108.30 (17)	O15—C52—C53	124.93 (19)
C11—C12—C15	114.00 (17)	O15—C52—C51	115.55 (18)
C12—C13—H13A	109.5	C53—C52—C51	119.52 (18)
C12—C13—H13B	109.5	C52—C53—C54	121.5 (2)
H13A—C13—H13B	109.5	C52—C53—H53	119.2
C12—C13—H13C	109.5	C54—C53—H53	119.2
H13A—C13—H13C	109.5	C53—C54—C49	119.5 (2)
H13B—C13—H13C	109.5	C53—C54—C58	118.10 (19)
C11—C14—H14A	109.5	C49—C54—C58	122.42 (18)
C11—C14—H14B	109.5	O17—C55—H55A	109.5
H14A—C14—H14B	109.5	O17—C55—H55B	109.5
C11—C14—H14C	109.5	H55A—C55—H55B	109.5
H14A—C14—H14C	109.5	O17—C55—H55C	109.5
H14B—C14—H14C	109.5	H55A—C55—H55C	109.5
C16—C15—C12	115.80 (17)	H55B—C55—H55C	109.5
C16—C15—H15A	108.3	O16—C56—H56A	109.5
C12—C15—H15A	108.3	O16—C56—H56B	109.5
C16—C15—H15B	108.3	H56A—C56—H56B	109.5
C12—C15—H15B	108.3	O16—C56—H56C	109.5
H15A—C15—H15B	107.4	H56A—C56—H56C	109.5
C21—C16—C17	119.7 (2)	H56B—C56—H56C	109.5
C21—C16—C15	121.89 (19)	O15—C57—H57A	109.5
C17—C16—C15	118.36 (19)	O15—C57—H57B	109.5
C18—C17—C16	121.22 (19)	H57A—C57—H57B	109.5
C18—C17—H17	119.4	O15—C57—H57C	109.5
C16—C17—H17	119.4	H57A—C57—H57C	109.5
O6—C18—C17	125.14 (19)	H57B—C57—H57C	109.5
O6—C18—C19	115.19 (18)	C54—C58—C59	116.76 (18)
C17—C18—C19	119.64 (19)	C54—C58—H58A	108.1
O5—C19—C20	119.61 (19)	C59—C58—H58A	108.1
O5—C19—C18	120.61 (18)	C54—C58—H58B	108.1
C20—C19—C18	119.59 (19)	C59—C58—H58B	108.1
O4—C20—C19	118.86 (19)	H58A—C58—H58B	107.3
O4—C20—C21	120.05 (18)	C62—C59—C58	112.21 (18)
C19—C20—C21	121.1 (2)	C62—C59—C60	114.31 (18)
C16—C21—C20	118.70 (19)	C58—C59—C60	113.24 (17)
C16—C21—C1	121.56 (19)	C62—C59—H59	105.4
C20—C21—C1	119.71 (19)	C58—C59—H59	105.4
O4—C22—H22A	109.5	C60—C59—H59	105.4
O4—C22—H22B	109.5	O21—C60—C61	109.15 (18)
H22A—C22—H22B	109.5	O21—C60—C63	105.61 (17)
O4—C22—H22C	109.5	C61—C60—C63	109.12 (18)
H22A—C22—H22C	109.5	O21—C60—C59	108.60 (17)
H22B—C22—H22C	109.5	C61—C60—C59	110.25 (18)
O5—C23—H23A	109.5	C63—C60—C59	113.93 (17)
O5—C23—H23B	109.5	C60—C61—H61A	109.5
H23A—C23—H23B	109.5	C60—C61—H61B	109.5
O5—C23—H23C	109.5	H61A—C61—H61B	109.5
H23A—C23—H23C	109.5	C60—C61—H61C	109.5
H23B—C23—H23C	109.5	H61A—C61—H61C	109.5
O6—C24—H24A	109.5	H61B—C61—H61C	109.5
O6—C24—H24B	109.5	C59—C62—H62A	109.5
H24A—C24—H24B	109.5	C59—C62—H62B	109.5
O6—C24—H24C	109.5	H62A—C62—H62B	109.5
H24A—C24—H24C	109.5	C59—C62—H62C	109.5
H24B—C24—H24C	109.5	H62A—C62—H62C	109.5
C30—C25—C26	118.26 (18)	H62B—C62—H62C	109.5
C30—C25—C41	122.52 (19)	C64—C63—C60	114.78 (18)
C26—C25—C41	119.08 (19)	C64—C63—H63A	108.6
C27—C26—O10	118.59 (18)	C60—C63—H63A	108.6
C27—C26—C25	121.77 (19)	C64—C63—H63B	108.6
O10—C26—C25	119.61 (18)	C60—C63—H63B	108.6
O9—C27—C26	120.04 (19)	H63A—C63—H63B	107.5
O9—C27—C28	120.07 (18)	C65—C64—C69	119.99 (19)
C26—C27—C28	119.64 (19)	C65—C64—C63	122.26 (18)
O8—C28—C29	124.65 (19)	C69—C64—C63	117.72 (19)
O8—C28—C27	116.10 (19)	C64—C65—C66	118.22 (18)
C29—C28—C27	119.22 (19)	C64—C65—C49	122.05 (19)
C28—C29—C30	121.3 (2)	C66—C65—C49	119.72 (19)
C28—C29—H29	119.3	O18—C66—C67	118.50 (19)
C30—C29—H29	119.3	O18—C66—C65	119.93 (18)
C25—C30—C29	119.72 (19)	C67—C66—C65	121.56 (19)
C25—C30—C34	122.30 (19)	O19—C67—C66	119.84 (19)
C29—C30—C34	117.94 (19)	O19—C67—C68	120.49 (18)
O8—C31—H31A	109.5	C66—C67—C68	119.44 (19)
O8—C31—H31B	109.5	O20—C68—C69	124.4 (2)
H31A—C31—H31B	109.5	O20—C68—C67	116.16 (19)
O8—C31—H31C	109.5	C69—C68—C67	119.44 (19)
H31A—C31—H31C	109.5	C68—C69—C64	121.3 (2)
H31B—C31—H31C	109.5	C68—C69—H69	119.4
O9—C32—H32A	109.5	C64—C69—H69	119.4
O9—C32—H32B	109.5	O18—C70—H70A	109.5
H32A—C32—H32B	109.5	O18—C70—H70B	109.5
O9—C32—H32C	109.5	H70A—C70—H70B	109.5
H32A—C32—H32C	109.5	O18—C70—H70C	109.5
H32B—C32—H32C	109.5	H70A—C70—H70C	109.5
O10—C33—H33A	109.5	H70B—C70—H70C	109.5
O10—C33—H33B	109.5	O19—C71—H71A	109.5
H33A—C33—H33B	109.5	O19—C71—H71B	109.5
O10—C33—H33C	109.5	H71A—C71—H71B	109.5
H33A—C33—H33C	109.5	O19—C71—H71C	109.5
H33B—C33—H33C	109.5	H71A—C71—H71C	109.5
C30—C34—C35	115.58 (17)	H71B—C71—H71C	109.5
C30—C34—H34A	108.4	O20—C72—H72A	109.5
C35—C34—H34A	108.4	O20—C72—H72B	109.5
C30—C34—H34B	108.4	H72A—C72—H72B	109.5
C35—C34—H34B	108.4	O20—C72—H72C	109.5
H34A—C34—H34B	107.4	H72A—C72—H72C	109.5
O14—C35—C38	108.95 (18)	H72B—C72—H72C	109.5
O14—C35—C36	108.52 (17)		
			
C7—O3—C2—C3	98.2 (2)	C34—C35—C36—C39	50.6 (2)
C7—O3—C2—C1	−82.4 (3)	C37—C36—C39—C40	45.8 (3)
C6—C1—C2—O3	−179.31 (18)	C35—C36—C39—C40	−85.7 (2)
C21—C1—C2—O3	1.5 (3)	C36—C39—C40—C41	93.5 (2)
C6—C1—C2—C3	0.1 (3)	C36—C39—C40—C45	−88.7 (2)
C21—C1—C2—C3	−179.17 (19)	C45—C40—C41—C42	1.7 (3)
C8—O2—C3—C2	−108.5 (2)	C39—C40—C41—C42	179.51 (19)
C8—O2—C3—C4	75.8 (3)	C45—C40—C41—C25	−174.68 (19)
O3—C2—C3—O2	1.9 (3)	C39—C40—C41—C25	3.1 (3)
C1—C2—C3—O2	−177.46 (19)	C30—C25—C41—C40	−61.6 (3)
O3—C2—C3—C4	177.69 (18)	C26—C25—C41—C40	114.1 (2)
C1—C2—C3—C4	−1.7 (3)	C30—C25—C41—C42	122.1 (2)
C9—O1—C4—C5	−10.2 (3)	C26—C25—C41—C42	−62.2 (3)
C9—O1—C4—C3	169.88 (19)	C46—O11—C42—C43	46.7 (3)
O2—C3—C4—O1	−2.9 (3)	C46—O11—C42—C41	−136.1 (2)
C2—C3—C4—O1	−178.58 (19)	C40—C41—C42—O11	−179.92 (18)
O2—C3—C4—C5	177.21 (18)	C25—C41—C42—O11	−3.5 (3)
C2—C3—C4—C5	1.5 (3)	C40—C41—C42—C43	−2.7 (3)
O1—C4—C5—C6	−179.6 (2)	C25—C41—C42—C43	173.8 (2)
C3—C4—C5—C6	0.3 (3)	C47—O12—C43—C42	−118.2 (2)
C4—C5—C6—C1	−1.9 (3)	C47—O12—C43—C44	68.6 (3)
C4—C5—C6—C10	179.24 (19)	O11—C42—C43—O12	5.5 (3)
C2—C1—C6—C5	1.7 (3)	C41—C42—C43—O12	−171.60 (18)
C21—C1—C6—C5	−179.1 (2)	O11—C42—C43—C44	178.75 (19)
C2—C1—C6—C10	−179.47 (19)	C41—C42—C43—C44	1.6 (3)
C21—C1—C6—C10	−0.3 (3)	C48—O13—C44—C45	−8.3 (3)
C5—C6—C10—C11	−86.6 (2)	C48—O13—C44—C43	170.97 (19)
C1—C6—C10—C11	94.6 (2)	O12—C43—C44—O13	−5.8 (3)
C6—C10—C11—C14	48.3 (3)	C42—C43—C44—O13	−178.96 (19)
C6—C10—C11—C12	−83.8 (2)	O12—C43—C44—C45	173.53 (19)
C14—C11—C12—O7	163.45 (18)	C42—C43—C44—C45	0.4 (3)
C10—C11—C12—O7	−66.0 (2)	O13—C44—C45—C40	177.9 (2)
C14—C11—C12—C13	43.0 (2)	C43—C44—C45—C40	−1.3 (3)
C10—C11—C12—C13	173.61 (18)	C41—C40—C45—C44	0.3 (3)
C14—C11—C12—C15	−79.3 (2)	C39—C40—C45—C44	−177.63 (18)
C10—C11—C12—C15	51.3 (2)	C55—O17—C50—C51	93.0 (2)
O7—C12—C15—C16	32.4 (2)	C55—O17—C50—C49	−88.6 (2)
C13—C12—C15—C16	149.82 (18)	C54—C49—C50—O17	−178.97 (18)
C11—C12—C15—C16	−86.6 (2)	C65—C49—C50—O17	0.6 (3)
C12—C15—C16—C21	94.2 (2)	C54—C49—C50—C51	−0.7 (3)
C12—C15—C16—C17	−85.6 (2)	C65—C49—C50—C51	178.9 (2)
C21—C16—C17—C18	−0.6 (3)	C56—O16—C51—C50	−114.1 (2)
C15—C16—C17—C18	179.11 (19)	C56—O16—C51—C52	70.7 (3)
C24—O6—C18—C17	19.7 (3)	O17—C50—C51—O16	3.1 (3)
C24—O6—C18—C19	−158.52 (19)	C49—C50—C51—O16	−175.26 (18)
C16—C17—C18—O6	−176.9 (2)	O17—C50—C51—C52	178.40 (18)
C16—C17—C18—C19	1.3 (3)	C49—C50—C51—C52	0.0 (3)
C23—O5—C19—C20	99.6 (3)	C57—O15—C52—C53	−6.6 (3)
C23—O5—C19—C18	−85.5 (3)	C57—O15—C52—C51	173.24 (18)
O6—C18—C19—O5	1.6 (3)	O16—C51—C52—O15	−4.8 (3)
C17—C18—C19—O5	−176.7 (2)	C50—C51—C52—O15	−179.91 (18)
O6—C18—C19—C20	176.6 (2)	O16—C51—C52—C53	175.10 (18)
C17—C18—C19—C20	−1.8 (3)	C50—C51—C52—C53	−0.1 (3)
C22—O4—C20—C19	74.9 (3)	O15—C52—C53—C54	−179.4 (2)
C22—O4—C20—C21	−103.9 (2)	C51—C52—C53—C54	0.7 (3)
O5—C19—C20—O4	−2.1 (3)	C52—C53—C54—C49	−1.3 (3)
C18—C19—C20—O4	−177.12 (19)	C52—C53—C54—C58	−179.38 (18)
O5—C19—C20—C21	176.7 (2)	C50—C49—C54—C53	1.3 (3)
C18—C19—C20—C21	1.7 (3)	C65—C49—C54—C53	−178.28 (19)
C17—C16—C21—C20	0.5 (3)	C50—C49—C54—C58	179.24 (18)
C15—C16—C21—C20	−179.2 (2)	C65—C49—C54—C58	−0.3 (3)
C17—C16—C21—C1	−177.6 (2)	C53—C54—C58—C59	−87.2 (2)
C15—C16—C21—C1	2.7 (3)	C49—C54—C58—C59	94.8 (2)
O4—C20—C21—C16	177.7 (2)	C54—C58—C59—C62	46.5 (3)
C19—C20—C21—C16	−1.0 (3)	C54—C58—C59—C60	−84.8 (2)
O4—C20—C21—C1	−4.1 (3)	C62—C59—C60—O21	165.15 (18)
C19—C20—C21—C1	177.1 (2)	C58—C59—C60—O21	−64.6 (2)
C2—C1—C21—C16	116.6 (2)	C62—C59—C60—C61	45.6 (2)
C6—C1—C21—C16	−62.6 (3)	C58—C59—C60—C61	175.81 (18)
C2—C1—C21—C20	−61.5 (3)	C62—C59—C60—C63	−77.5 (2)
C6—C1—C21—C20	119.3 (2)	C58—C59—C60—C63	52.7 (2)
C33—O10—C26—C27	79.2 (2)	O21—C60—C63—C64	31.7 (2)
C33—O10—C26—C25	−98.7 (2)	C61—C60—C63—C64	148.91 (19)
C30—C25—C26—C27	1.0 (3)	C59—C60—C63—C64	−87.4 (2)
C41—C25—C26—C27	−174.8 (2)	C60—C63—C64—C65	95.4 (2)
C30—C25—C26—O10	178.85 (18)	C60—C63—C64—C69	−82.5 (2)
C41—C25—C26—O10	3.0 (3)	C69—C64—C65—C66	−0.3 (3)
C32—O9—C27—C26	89.7 (3)	C63—C64—C65—C66	−178.14 (19)
C32—O9—C27—C28	−96.0 (2)	C69—C64—C65—C49	178.6 (2)
O10—C26—C27—O9	−3.6 (3)	C63—C64—C65—C49	0.8 (3)
C25—C26—C27—O9	174.21 (18)	C50—C49—C65—C64	119.6 (2)
O10—C26—C27—C28	−177.96 (18)	C54—C49—C65—C64	−60.9 (3)
C25—C26—C27—C28	−0.1 (3)	C50—C49—C65—C66	−61.5 (3)
C31—O8—C28—C29	17.8 (3)	C54—C49—C65—C66	118.0 (2)
C31—O8—C28—C27	−160.08 (19)	C70—O18—C66—C67	82.9 (2)
O9—C27—C28—O8	1.8 (3)	C70—O18—C66—C65	−96.0 (2)
C26—C27—C28—O8	176.14 (19)	C64—C65—C66—O18	−179.24 (18)
O9—C27—C28—C29	−176.21 (19)	C49—C65—C66—O18	1.8 (3)
C26—C27—C28—C29	−1.9 (3)	C64—C65—C66—C67	1.9 (3)
O8—C28—C29—C30	−174.84 (19)	C49—C65—C66—C67	−177.08 (19)
C27—C28—C29—C30	3.0 (3)	C71—O19—C67—C66	97.4 (2)
C26—C25—C30—C29	0.0 (3)	C71—O19—C67—C68	−88.1 (3)
C41—C25—C30—C29	175.8 (2)	O18—C66—C67—O19	−5.6 (3)
C26—C25—C30—C34	−177.49 (19)	C65—C66—C67—O19	173.29 (19)
C41—C25—C30—C34	−1.8 (3)	O18—C66—C67—C68	179.85 (18)
C28—C29—C30—C25	−2.1 (3)	C65—C66—C67—C68	−1.3 (3)
C28—C29—C30—C34	175.57 (18)	C72—O20—C68—C69	19.1 (3)
C25—C30—C34—C35	96.9 (2)	C72—O20—C68—C67	−159.31 (19)
C29—C30—C34—C35	−80.7 (2)	O19—C67—C68—O20	3.0 (3)
C30—C34—C35—O14	35.0 (2)	C66—C67—C68—O20	177.51 (19)
C30—C34—C35—C38	151.82 (19)	O19—C67—C68—C69	−175.47 (19)
C30—C34—C35—C36	−84.2 (2)	C66—C67—C68—C69	−1.0 (3)
O14—C35—C36—C37	162.56 (18)	O20—C68—C69—C64	−175.8 (2)
C38—C35—C36—C37	43.0 (2)	C67—C68—C69—C64	2.6 (3)
C34—C35—C36—C37	−79.6 (2)	C65—C64—C69—C68	−1.9 (3)
O14—C35—C36—C39	−67.2 (2)	C63—C64—C69—C68	176.02 (19)
C38—C35—C36—C39	173.16 (18)		

### Hydrogen-bond geometry (Å, °)

**Table:**

*D*—H···*A*	*D*—H	H···*A*	*D*···*A*	*D*—H···*A*
O7—H7···O2^i^	0.84	2.10	2.836 (2)	146
O14—H14···O16^ii^	0.84	2.14	2.889 (2)	149
O21—H21···O12^iii^	0.84	2.00	2.793 (2)	156
C11—H11···O3^i^	1.00	2.58	3.469 (3)	148
C37—H37A···O8^iv^	0.98	2.60	3.490 (3)	151
C37—H37C···O5^v^	0.98	2.58	3.498 (3)	157
C38—H38B···O18^ii^	0.98	2.51	3.389 (3)	150
C24—H24B···Cg1^vi^	0.98	2.80	3.710 (3)	156
C31—H31B···Cg2^vii^	0.98	2.71	3.653 (3)	181
C72—H72B···Cg3^vi^	0.98	2.94	3.894 (3)	164

Symmetry codes: (i) −*x*+1, *y*−1/2, −*z*+3/2; (ii) *x*+1/2, −*y*+3/2, −*z*+1; (iii) *x*−1/2, −*y*+1/2, −*z*+1; (iv) *x*+1/2, −*y*+3/2, −*z*+2; (v) −*x*+2, *y*+1/2, −*z*+3/2; (vi) *x*+1/2, −*y*+1/2, −*z*+1; (vii) *x*−1/2, −*y*+3/2, −*z*+2.
